# Effects of nonlinear thermal radiation on the efficiency of building integrated photovoltaic systems with nanofluid cooling

**DOI:** 10.1371/journal.pone.0304685

**Published:** 2024-06-20

**Authors:** Nacer Badi, Ali Hatem Laatar, Anouar Ben Mabrouk, Abdulrhman M. Alsharari, Saleh A. Alghamdi, Hani Albalawi

**Affiliations:** 1 Department of Physics, Faculty of Sciences, University of Tabuk, Tabuk, Saudi Arabia; 2 Renewable Energy & Environmental Technologies Research Center, University of Tabuk, Tabuk, Saudi Arabia; 3 Laboratory of Energetics and Thermal and Mass Transfer (LR01ES07), Faculty of Sciences of Tunis, University of Tunis El Manar, Tunis, Tunisia; 4 Department of Mathematics, Faculty of Sciences, University of Tabuk, Tabuk, Saudi Arabia; 5 Electrical Engineering Department, Faculty of Engineering, University of Tabuk, Tabuk, Kingdom of Saudi Arabia; Beijing University of Technology, CHINA

## Abstract

The nonlinear effects of thermal radiation on the free convection flow of certain nanofluids along a heated wall are studied numerically using an original finite-difference method. Nanofluids are used to improve the performance of flat and curved integrated photovoltaic modules. The partial differential equations governing the flow are difficult to solve due to the strong non-linearity of the radiative term. In contrast to previous studies, the problem is solved directly without linearization by Rosseland’s nonlinear approximation. The proposed numerical method is validated with results from the literature. The effects of nonlinearity and various physical parameters such as time, volume fraction and radiation parameter on the velocity, temperature, Nusselt number and skin friction coefficient of the CuO-water nanofluid are analyzed and presented graphically. A comparative study between the solutions given by the linear and non-linear problems reveals that Rosseland’s linear approximation is no longer valid when the effect of thermal radiation is significant. On the other hand, the non-linear model better reflects the physical phenomena involved in the cooling process. Finally, a comparison of the performance of five nanofluids (CuO, Ag, Al_2_O_3_, Cu and TiO_2_ in water) shows that the Cu-water nanofluid performs best, with a high heat transfer rate and low shear stresses.

## 1. Introduction

Solar energy is the most common renewable resource on earth and can be converted into electricity and heat using solar panels and thermal systems. However, the efficiency of solar modules is low, only 15–20% of solar radiation is converted into electrical energy and the rest is lost as heat [1, 2]. In addition, the increase in the temperature of photoelectric cells can lead to a reduction in the electrical efficiency of the system, as well as the risk of overheating and failure in photoelectric panels [3].

Various methods have been developed to regulate the temperature of PV modules and utilize the excess heat generated by the PV cells. One such solution is the solar photovoltaic/thermal (PV/T) system, which includes a thermal collector added to the PV module to recover the excess heat generated by the PV cells. This system has the advantage of simultaneously producing thermal and electrical energy, lowering the temperature of the PV cells, and increasing their electrical efficiency [4, 5].

Building Integrated Photovoltaic Thermal (BIPVT) systems are an advanced technology that integrates Photovoltaic Thermal (PVT) systems into buildings. This integration allows buildings to harness solar energy, reducing their energy consumption and carbon footprint. BIPVT systems are highly efficient as they generate both electricity and heat simultaneously. As a result, they help reduce greenhouse gas emissions, making them a sustainable and eco-friendly energy solution for buildings. They are an attractive option for sustainable building design because they can be seamlessly integrated into a building’s architecture. BIPVT systems offer architects and building designers the opportunity to enhance the aesthetic appearance of buildings. With flat and curved PV modules, architects can create stunning designs by seamlessly integrating solar panels into building facades, roofs, or shading devices. The use of rounded geometric shapes in modern and traditional orient architecture, such as domes, arches, and curved roofs, can be enhanced by curved PV modules, while classical PV modules can be used on flat surfaces. The integration of both types of PV modules into buildings not only provides renewable energy but also improves the overall appearance of the building.

PV/T hybrid systems commonly use water and air as heat transfer fluids, but their low thermal conductivity makes them less efficient. To improve heat transfer in these systems, we need to use working fluids with better thermophysical properties. Nanofluids are a promising option. They have been shown to significantly improve the heat transfer characteristics in PV/T systems compared to conventional fluids.

Nanofluids are a type of fluid that contains very small particles, typically less than 100 nm in size, suspended in a base fluid. Despite their small size, the nanoparticles have a large surface area, which increases the thermal conductivity of the fluid and improves its heat transfer capabilities. Nanofluids have a wide range of potential applications, including engineering and solar energy systems. The concept of nanofluids was first introduced by Choi and Eastman [6] in 1995 for the purpose of improving heat transfer.

For two decades, many works have been carried out around the introduction of nanofluids in the cooling methods of PV/T systems. The results obtained show a significant improvement in the electrical and thermal performances of these systems.

In a study by Ghadiri et al. [7], experiments were conducted to investigate the use of Fe_3_O_4_-water ferrofluid to cool a photovoltaic thermal system (PVT). Different concentrations of the nanofluid were tested, ranging from 1 to 3 wt%, and the PV cells were exposed to different levels of solar radiation (600 and 1100 w/m^2^) to evaluate the effect on system efficiency and exergy rate. Constant and variable magnetic fields were applied to the ferrofluid to improve its performance. The results indicate that the overall efficiency of the system can reach up to 79% when using a 50 Hz alternating magnetic field and a 3% ferrofluid concentration.

Khanjari et al. [8] conducted a numerical study on the use of Ag-water and alumina-water nanofluids to cool a PVT system. They evaluated the effects of varying the inlet fluid velocity and volume fraction on heat transfer enhancement and efficiency. The study found that increasing the volume fraction of nanoparticles in the nanofluids led to a 12% and 43% improvement in the heat transfer coefficient for alumina-water and Ag-water nanofluids, respectively. Additionally, when the volume fraction was increased from 1 to 10%, the thermal efficiency of water-Al_2_O_3_ and water-Ag nanofluid improved by 3% and 10%, respectively. Overall, the electrical and thermal performance of water-Ag nanofluid was found to be better than that of alumina-water nanofluid.

Silicon carbide SiC nanoparticles were used by Al-Waeli et al. [9] to improve the performance of the PVT system. In these experiments, they varied the concentrations of the nanoparticles from 1 to 4 wt% to study the thermophysical properties of the nanofluid. The results indicate that the solution is very stable and is suitable for long-term uses. Additionally, it was observed that the thermal conductivity was increased by more than 8%. Furthermore, the study revealed that a 3 wt% SiC nanofluid can improve the thermal efficiency by 100% and the electrical efficiency by 24%.

To compare the performance of three PV/T system cooling techniques, Al-Waeli et al. [10] performed a series of tests using cooling pipes through which water, nanofluids, and nano-PCMs flow. They then used an artificial neural network to analyze the results and found that the nanofluid/nano-PCM technique was the most effective, resulting in a 72% improvement in thermal efficiency and a 5.25% increase in electrical efficiency. The study also revealed that thermal efficiency is primarily affected by ambient temperature, while electrical efficiency is influenced by both solar irradiation and ambient temperature.

Sangeetha et al. [11] conducted an experiment to examine the impact of three types of nanoparticles (MWCNT, Al_2_O_3_, and CuO) on the thermal and electrical performance of PV/T systems. They analyzed various thermophysical properties of fluids with different volume fractions of nanoparticles ranging from 0 to 5% suspended in water. The tests show that the use of nanofluids (MWCNT, Al_2_O_3,_ and CuO with water) reduces the temperature of PV cells by 19% and improves their average electrical efficiency by more than 52%. Among the three types of nanoparticles tested, a multi-walled carbon nanotube (MWCNT) performed the best, with an electrical efficiency of 60%.

In a study by Kazem et al. [12], the cooling of a PV/T system using single-walled carbon nanotube nanofluids (SWCNTs) was experimentally examined. The fluid mixture consisted of 75% water and 25% ethylene glycol, along with a surfactant. Various volume fractions of SWCNTs (0.1%, 0.5%, 1.0%, and 2.0%) were tested to observe their effect on thermophysical properties. The results showed that incorporating SWCNTs decreased the temperature of the PV module by 18% and increased the generated electrical power by 11.7%, leading to a 25.2% improvement in the electrical efficiency of the PV/T system. This resulted in an overall efficiency increase of 11% to 71% compared to a conventional PV system.

Anderson et al. [13] designed a hybrid system that uses solar energy to produce hydrogen by integrating an electrolyzer and a PV/T system. The electrical energy generated by the PV/T system is used to produce hydrogen by electrolysis, and a cooling system using nanofluids has been implemented to improve thermal and electrical performance. The system was evaluated using MWCNT and Fe_2_O_3_ nanoparticles, and the best hydrogen production was obtained at a mass flow rate of 0.01 kg/s between 12:15 and 13:00. Khodadadi et al. [14] developed a 3D model to evaluate the efficiency of a PVT/PCM system using nanoparticle-enhanced phase change materials (NEPCM) with different types of nanofluids. They experimented with different concentrations of nanoparticles, such as SiC, ZnO, MWCNT, Al_2_O_3_, Cu, and Ag, in a mixture of water and PCM, and examined the impact of solar radiation intensity and flow rate on system performance. They also compared the integration of a thermoelectric module (TE) into the PV/T system with other PVT, PVT/PCM, PVT-TE, and PVT-TE/PCM hybrid collector systems. The analysis revealed a 7.06% decrease in the average PV temperature and a 35.13% decrease in the average output temperature. In a recent study, Wang et al. [15] proposed a new type of photovoltaic/thermal roof using airflow and transparent cover to improve the overall performance of a building-integrated photovoltaic/thermal system. The study compared glazed and unglazed systems, and a numerical model was built and validated based on the experimental data. The glazed system demonstrated superior thermal efficiency, capable of heating a connected room with warm air, while the unglazed system was slightly more electrically efficient. The research also examined the influence of various factors, such as the tilt angle of the bottom plate and the extinction coefficient of the roofing material. Kumar and Dhiman [16] conducted a study to improve the performance and lifetime of PV modules through cooling. The authors used statistical models and response surface methodology to examine the performance parameters of a recirculating photovoltaic thermal system, including thermal efficiency, electrical efficiency, PV module temperature, thermal energy gain, and net electrical power. They found that mass flow rate was the most important factor influencing the thermal and electrical performance of the PVT system. Kazemian et al. [17] presented a novel design of a photovoltaic thermal system with a solar thermal collector enhancer, which partially covers the absorber plate with photovoltaic cells to increase energy conversion and efficiency. A three-dimensional model was developed to analyze the impact of different glazing arrangements on system performance, as well as other parameters such as tilt angle and dust accumulation. A comparative study was also conducted with stand-alone solar thermal and photovoltaic systems, which showed that the proposed system has a higher overall power output and a shorter payback time.

Previous studies have shown that, under certain conditions, the use of nanofluids can greatly improve the thermal and electrical performance of photovoltaic and thermal systems. However, it is important that the flow remains laminar but with a mass flow rate approaching that of a turbulent regime. In addition, increasing the concentration of nanoparticles can improve efficiency, but it should not exceed a certain limit, as the efficiency will decrease due to particle agglomeration [18]. Studies on heat transfer between a PV/T solar system and a nanofluid bring us back to the fundamental problem of natural convection along an infinite plate. This type of flow has been widely investigated due to its various engineering applications, including the use of nanofluids to cool PV panels. However, despite numerous studies on the subject, the impact of thermal radiation on nanofluid flow along an infinite plate is not fully understood. Thermal radiation plays a significant role in heat transfer when there is a large temperature difference between the wall and the surrounding fluid. The presence of thermal radiation alters the heat transfer modes and changes the structure of the thermal boundary layer. The incorporation of thermal radiative effects allows for more accurate predictions of the flow and heat exchanges.

In this type of problem, the radiative heat transfer has been neglected or not given enough consideration, in comparison to convective or conductive effects. However, it’s important to accurately model thermal radiation as it plays a significant role in heat exchange. Various approaches have been proposed in the literature to account for thermal radiation, including the Rosseland diffusion model, the composite radiosity model, and the six-flux radiation formulation. The linearized form of the Rosseland approximation is commonly used by many authors to model the radiative term in the energy equation. Furthermore, some researchers have developed analytical solutions for heat transfer problems involving thermal radiation using fractional derivatives and Laplace transform techniques. However, these methods can only be applied when the radiative term in T^4^ is linearized, which assumes a small temperature difference between the wall and ambient fluid. The linearized Rosseland approximation has facilitated solving of many challenging radiative problems and enabled the analysis of intricate thermal systems. However, these solutions are not accurate as they do not consider the nonlinear aspect of the radiative term in the energy equation.

Using the Caputo time-fractional derivative, Fetecau et al. [19] studied the free convection of two water-based fractional nanofluids over an infinite vertical plate with thermal radiation. They discussed the impact of the nanoparticles’ volume fraction, thermal radiation parameter, and fractional parameter on the velocity, temperature, skin friction coefficient, and heat transfer rate. Aman et al. [20] addressed heat and mass transfer in graphene nanofluids using fractional derivatives. They obtained analytical solutions for temperature, concentration, velocity, and Nusselt number through Laplace transform. They also compared the results with numerical solutions given by the finite difference method for velocity, temperature, concentration, and Nusselt number. Raza et al. [21] investigated the heat exchange of Casson nanoparticles near an infinite vertical surface using the Atangana-Baleanu time-fractional derivative method. They solved the equations for convective flow using Laplace transformation and examined the impact of various parameters on temperature and velocity profiles. The study showed that the Atangana-Baleanu fractional time derivative provides temperature and velocity profiles with more decreasing aspects than those of the Caputo fractional derivative. Rehman et al. [22] developed a fractional model to study the time-dependent natural convective flow of Maxwell fluid over an isothermal vertical plate that extends to infinity. They used the fractional Prabhakar operator with a Mittag-Leffler kernel in the constitutive equations and employed the Laplace transform to find exact solutions for the dimensionless velocity and concentration. The study analyzed and discussed the impact of various physical parameters on the performance of the system. The results indicate that Maxwell fluids move faster than viscous fluids in both the fractional and classical cases. In many situations, the temperature difference between the fluid and the wall is large and the linear Rosseland approximation becomes unreliable. The radiative term then plays a crucial role in heat transfer and affects the flow structure and thermal boundary layer thickness. The introduction of nonlinear radiative effects in the energy equation makes the governing equations highly nonlinear which requires more advanced modeling [23]. The nonlinear Rosseland approximation, proposed by Pantokratoras and Fang [24], provides a more accurate solution to these equations. Recently, the effects of thermal radiation have been analyzed by some authors using the nonlinear Rosseland approximation. Jamshed et al. [25] investigated the flow of a hybrid nanofluid over a porous stretching surface. The heat transfer characteristics of two types of nanofluids were compared by considering the effect of thermal radiation, the shape of the nanoparticles, and thermal conductivity. Acharya et al. [26] studied the thermodynamic performance of a viscous nanofluid flow over a non-isothermal wedge. They analyzed the evolution of certain thermodynamic parameters under the effect of nonlinear radiation and activation energy. They also examined how the Reynolds number affected the increase of entropy generation number. Gamal El-Din A Azzam [27] studied the effects of radiation on mixed free-forced convective flow MHD past a moving semi-infinite vertical plate. The energy equation’s radiative term was modeled using the nonlinear Rosseland approximation, which accounts for the large temperature gap between the fluid and plate. The study aimed to evaluate the effect of magnetic field and radiation on heat transfer rate, skin friction, temperature patterns, and velocity profiles.

Ashraf et al. [28] conducted a numerical study to explore the impact of radiation on the steady mixed convection boundary layer flow of a viscous, incompressible, electrically conductive fluid along a semi-infinite magnetized vertical porous plate. They also examined the influence of radiation on the fluctuating hydromagnetic natural convection flow of an electrically conductive fluid past a magnetized vertical plate [29]. In addition, they studied the combined effects of radiation and hydromagnetism on the natural convection flow of a viscous, incompressible, electrically conductive fluid along a magnetized permeable vertical plate [30]. Their analysis included careful examination of the effect of various physical parameters on the skin friction coefficient, Nusselt number, current density and magnetic intensity coefficient. Ashraf et al. have extended their work to more complex geometries. They numerically studied the natural convective flow of the nanofluid boundary layer around different stations of a sphere and in the plume region above the sphere [31]. They analyzed the boundary layer characteristics near the sphere surface and the eruption of boundary layer fluid into the plume above the sphere.

Recently, Zahir et al. [32] conducted a study to investigate the effects of nonlinear thermal radiation and homogeneous-heterogeneous reactions on the peristaltic flow of a Johnson-Segalman fluid in a curved channel under the influence of a radial magnetic field. The main objectives were to analyze the properties of peristaltic flow and evaluate the factors affecting fluid behavior.

In the literature, we find numerous studies devoted to the flow of fluids with thermal radiation for various applications and in very different fields. This confirms the importance of this parameter in determining the flow behavior of certain fluids [32–39].

The unsteady natural convection flow of a nanofluid in the presence of radiation is modeled by a system of highly nonlinear partial differential equations. This system is difficult to solve analytically and numerically due to the strong non-linearity of the radiative term in the thermal energy equation. Most approaches proposed in the literature circumvent the difficulty of nonlinearity by linearizing this term, even though the phenomenon of thermal radiation is intrinsically nonlinear. As a result, solutions to the linearized problem do not faithfully reflect physical reality and are only valid for very restricted domains. Thus, to obtain accurate solutions valid for large domains, it is necessary to solve the nonlinear problem directly without modification.

The aim of this work is to numerically study the nonlinear effects of thermal radiation on the free convection flow of some nanofluids along an infinite vertical plate. The final objective is to improve cooling techniques for photovoltaic modules and PV/T systems using nanofluids. Rosseland’s nonlinear approximation [40] is applied to describe radiative heat transfer in the energy equation. The set of non-dimensional governing equations is solved numerically by an original approach based on the finite difference method without linearizing the radiative term. The finite-difference approximation scheme used is stable and converges with second-order accuracy. The proposed numerical method is first validated by comparing the Nusselt number and skin friction coefficient with the numerical results presented by Fetecau et al. [19].

The effects of various physical parameters such as time, volume fraction and radiation parameters on the velocity, temperature, heat transfer rates and shear stresses of the CuO-water nanofluid are analyzed and presented graphically.

A comparative study is carried out to evaluate the differences between the numerical solutions obtained by applying the linear or non-linear Rosseland approximation, in order to clarify the effects of linearizing the radiative term on the quality of the computed solutions. Finally, the performance of five types of nanofluids containing CuO, Ag, Al_2_O_3_, Cu and TiO_2_ is compared using the average Nusselt number Nu and the average skin friction coefficient C_f_ as a benchmark.

## 2. Methods

**Fig 1:**
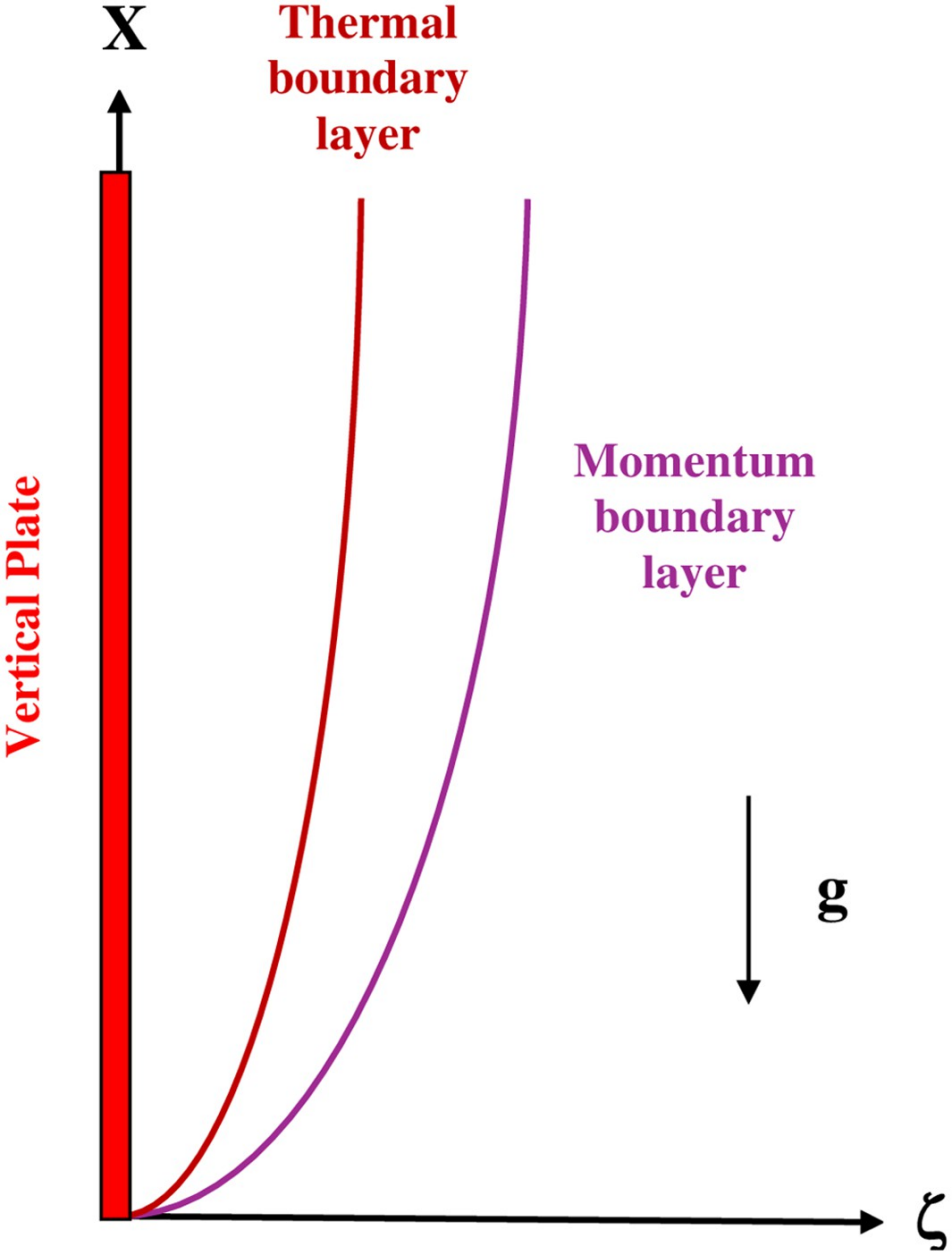
Physical configuration of the problem.

Consider the unsteady flow of a nanofluid along an infinite vertical plate. The flow is along the x-axis directed upwards, while the *ζ*-axis is normal to the plate. Initially, the fluid and the plate are at rest and are at the ambient temperature *T*_*∞*_. At t>0, the plate is heated and maintained at a constant temperature T_w_ above room temperature (T_w_>*T*_*∞*_). This creates a natural convection upward flow, driven by thermal buoyancy. As a result, a nanofluid boundary layer flow develops along the plate, characterized by thermal and momentum boundary layers. Fig 1 shows schematically the studied physical configuration with the coordinate system.

The fluid is assumed to be gray, which means that it is capable of absorbing and emitting radiation but not scattering it. In addition, the radiative heat flux is mainly in the direction orthogonal to the plate. The fluid is a water-based nanofluid with five types of nanoparticles, and it is assumed that the nanoparticles are in thermal equilibrium with the base fluid. As the plate is infinitely long, the temperature and velocity fields are only functions of *ζ* and t.

The thermophysical properties of water and nanoparticles are given in Table 1.

**Table 1:** The thermophysical properties [41].

Water/*Nanoparticles*	*ρ(kg/m* ^*3*^ *)*	*C*_*p*_ *(J/kg K)*	*k(W/mK)*	*β10* ^*5*^ *(K* ^*−1*^ *)*
Pure water (*H*_2_*O*)	997.1	4179	0.613	21
Copper (Cu)	8933	385	401	1.67
Copper oxide (CuO)	6320	531.8	76.5	1.80
Silver (Ag)	10500	235	429	1.89
Alumina (*Al*_2_*O*_3_)	3970	765	40	0.85
Titanium Oxide (*TiO*_2_)	4250	686.2	8.9538	0.9

In this study, we adopt the single-phase model for nanofluids, which assumes the existence of a local thermal equilibrium between the base fluid and the suspended nanoparticles, so that there is no slip between them. In the single-phase model, the nanofluid is considered as a homogeneous mixture, where the nanoparticles are assumed to be uniformly distributed in the base fluid. Consequently, the effects of nanoparticles on fluid properties (viscosity, thermal conductivity and density) are incorporated into those of the nanofluid. This also assumes that nanoparticles do not agglomerate.

According to the model proposed by Tiwari and Das [42] using the Boussinesq and boundary layer approximations, the unsteady nanofluid flow is governed by the equations of momentum and thermal energy given by the following system:

$$\left\{\begin{array}{l} \rho_{nf}\frac{\partial V}{\partial t}=\mu_{nf}\frac{\partial^{2}V}{\partial \zeta^{2}}+g{\left(\rho \beta_{T}\right)}_{nf}\left(T-T_{\infty }\right),\\ {\left(\mu C_{p}\right)}_{nf}\frac{\partial T}{\partial t}=K_{nf}\frac{\partial^{2}T}{\partial \zeta^{2}}-\frac{\partial Q_{r}}{\partial \zeta } \end{array}\right.$$ (1)

where *T* is the temperature of the nanofluid, V is the velocity along the vertical direction, (*ρC*_*p*_) _*nf*_ is the heat capacitance of the nanofluid, *K*_*nf*_ is the thermal conductivity of the nanofluid, *ρ*_*nf*_ is the density of the nanofluid, *μ*_*nf*_ is the dynamic viscosity of the nanolfuid, *β*_*nf*_ is the thermal expansion coefticient of the nanolfuid, *g* is the acceleration of gravity, and *Q*_*r*_ is the radiative heat flux.

The density *ρ*_*nf*_, thermal expansion coefficient (*ρβ*)_*nf*_, and heat capacitance (*ρC*_*p*_)_*nf*_ of nanofluids are expressed by the relations:

$$\rho_{nf}=\left(1-\varphi \right)\rho_{f}+\varphi \rho_{s}$$

$${\left(\rho \beta \right)}_{nf}=\left(1-\varphi \right)({\rho \beta )}_{f}+\varphi {\left(\rho \beta \right)}_{s}$$

$${\left(\rho C_{p}\right)}_{nf}=\left(1-\varphi \right)({\rho C_{p})}_{f}+\varphi {\left(\rho C_{p}\right)}_{s}$$

where *φ* is the nanoparticles volume fraction, *ρ*_*f*_ and *ρ*_*s*_ are the density of the base fluid and nanoparticles, the volumetric coefficient of thermal expansions of nanoparticles and base fluid are denoted by *β*_*s*_ and *β*_*f*_ respectively. (*C*_*p*_)_*s*_ and (*C*_*p*_)_*f*_ are the specific heat capacities of nanoparticles and base fluid at constant pressure.

The radiative heat flux *Q*_*r*_ is expressed by:

$$Q_{r}=-\frac{4\sigma_{SB}}{3K_{ma}}\frac{\partial T^{4}}{\partial \zeta }$$ (2)

using the Rosseland approximation [27]. Here *σ*_*SB*_ is the Stefan-Boltzman constant, and *K*_*ma*_ is the mean absorption coefficient. If the temperature difference between the free stream and the fluid is small, the nonlinear term *T*^4^ can be approximated by:

$$T^{4}=-4T_{\infty }^{3}T-3T_{\infty }^{4}$$ (3)

using Taylor expansion. Pantokratoras and Fang [23] proposed for a boundary layer flow over a horizontal flat plate to evaluate the radiative heat flux by:

$$Q_{r}=-\frac{16\sigma_{SB}}{3K_{ma}}\frac{\partial }{\partial \zeta }\left(T^{3}\frac{\partial T}{\partial \zeta }\right)$$ (4)

This last formula is a nonlinear extension of the Rosseland approximation valid for both small and large temperature differences between the plate and the ambient fluid.

By substituting (4) into the system (1) we obtain the following system (*T*, *V*)

$$\left\{\begin{array}{l} {\left(\rho C_{p}\right)}_{nf}\frac{\partial T}{\partial t}=K_{nf}\frac{\partial^{2}T}{\partial \zeta^{2}}+\frac{16\sigma_{SB}}{3K_{ma}}\frac{\partial }{\partial \zeta }\left(T^{3}\frac{\partial T}{\partial \zeta }\right)\\ \rho_{nf}\frac{\partial V}{\partial t}=\mu_{nf}\frac{\partial^{2}V}{\partial \zeta^{2}}+g{\left(\rho \beta_{T}\right)}_{nf}\left(T-T_{\infty }\right) \end{array}\right.$$ (5)

The problem above will be taken under the following initial boundary value conditions,

$$\left\{\begin{array}{l} V\left(0,\zeta \right)=0\;\text{and}\;T\left(0,\zeta \right)=T_{\infty },\;\forall \zeta >0\\ V\left(t,0\right)=0\;\text{and}\;T\left(t,0\right)=T_{w},\;\forall t>0\\ V\left(t,\zeta \right)\to 0\;\text{and}\;T\left(t,\zeta \right)\to 0\;\text{as}\;\zeta \to \infty ,\forall t>0 \end{array}\right.$$ (6)

Denote

$$L={\left(\frac{v_{f}^{2}}{g{\left(\beta_{T}\right)}_{f}\left(T_{w}-T_{\infty }\right)}\right)}^{\frac{1}{3}}$$

and consider the dimensionless variables

$$t^{*}=\frac{\nu_{f}}{L^{2}}t,\;\zeta^{*}=\frac{\zeta }{L},\;V^{*}=\frac{L}{v_{f}}V\;\text{and}\;{\;T}^{*}=\frac{T-T_{\infty }}{T_{w}-T_{\infty }}$$

Denote also

$$\begin{array}{c} a_{1}=\frac{\mu_{nf}}{\rho_{nf}v_{f}}=\frac{1}{(1-\phi {)}^{2.5}}\frac{1}{1-\phi +\phi \frac{\rho_{s}}{\rho_{f}}},\\ a_{2}=\frac{g{\left(\rho \beta_{T}\right)}_{nf}L^{3}}{\rho_{nf}v_{f}^{2}}\left(T_{w}-T_{\infty }\right)=\frac{1-\phi +\phi \frac{{\left(\rho \beta_{T}\right)}_{s}}{{\left(\rho \beta_{T}\right)}_{f}}}{1-\phi +\phi \frac{\rho_{s}}{\rho_{f}}} \end{array}$$

and

$$a_{3}=\frac{{\left(\rho C_{p}\right)}_{nf,}v_{f}}{K_{nf,}}=\frac{1}{Pr}\frac{\frac{K_{nf}}{K_{f}}}{1-\phi +\phi \frac{{\left(\rho C_{p}\right)}_{s}}{{\left(\mu C_{p}\right)}_{f}}},$$

and

$$a_{4}=\frac{16\sigma_{SB}{\left(T_{w}-T_{\infty }\right)}^{3}}{3K_{ma}{\left(\rho C_{p}\right)}_{nf}v_{f}}=\frac{Nr}{Pr}\frac{\frac{K_{11j}}{K_{f}}}{1-\phi +\phi \frac{{\left(\rho C_{p}\right)}_{s}}{{\left(\rho C_{p}\right)}_{f}}}=Nra_{3}$$

where

$$\begin{array}{c} Nr=\frac{16\sigma_{SB}{\left(T_{w}-T_{\infty }\right)}^{3}}{3K_{nf}K_{ma}},\\ \frac{K_{nf}}{K_{f}}=\frac{K_{s}+2K_{f}-2\phi \left(K_{f}-K_{s}\right)}{K_{s}+2K_{f}+\phi \left(K_{f}-K_{s}\right)}. \end{array}$$

We deduce the following nonlinear system from (1)

$$\left\{\begin{array}{rr} \frac{\partial V^{*}}{\partial t^{*}} & =a_{1}\frac{\partial^{2}V^{*}}{\partial^{2}\zeta^{*2}}+a_{2}T^{*}\\ \frac{\partial T^{*}}{\partial t^{*}} & =a_{3}\frac{\partial^{2}T^{*}}{\partial^{2}\zeta^{*2}}+a_{4}\frac{\partial }{\partial \zeta }\left(T^{*3}\frac{\partial T^{*}}{\partial \zeta }\right) \end{array}\right.$$ (7)

Without loss of the generality, we will omit the upper script⋆ and consider in the rest of the paper the following problem

$$\left\{\begin{array}{rr} \frac{\partial V}{\partial t} & =a_{1}\frac{\partial^{2}V}{\partial \zeta^{2}}+a_{2}T\\ \frac{\partial T}{\partial t} & =a_{3}\frac{\partial^{2}T}{\partial \zeta^{2}}+a_{4}\frac{\partial }{\partial \zeta }\left(T^{3}\frac{\partial T}{\partial \zeta }\right) \end{array}\right.$$ (8)

The problem (8) will be considered under the initial/boundary conditions

$$\left\{\begin{array}{l} V(0,\zeta )=0\;\text{and}\;T(0,\zeta )=1,\forall \zeta >0\\ V(t,0)=0\;\text{and}\;T(t,0)=0,\forall t>0\\ V(t,\zeta )\to 0\;\text{and}\;T(t,\zeta )\to 0\;\text{as}\;\zeta \to \infty ,\forall t>0 \end{array}\right.$$ (9)

The Nusselt number N_u_ and the skin friction coefficient C_f_ are the physical quantities relevant to engineering applications. They are defined as follows using dimensional variables:

$$N_{u}=\frac{Lq_{w}}{K_{f}\left(T_{w}-T_{\infty }\right)},\;\text{with}\;q_{w}=-{\left.K_{nf}\frac{\partial T}{\partial y}\right|}_{y=0}.$$

The Skin friction coefficient C_f_ is given by using the dimensional variables:

$$C_{f}=\frac{\tau_{w}}{\rho_{f}{\left(v_{f}/L\right)}^{2}},\;\text{with}\;\tau_{w}={\left.\mu_{nf}\frac{\partial V}{\partial y}\right|}_{y=0}$$

*τ*_*w*_ is skin friction or the shear stress.

On the other hand, using the dimensionless variables they are given by:

$$N_{u}=-{\left.\frac{K_{nf}}{K_{f}}\frac{\partial T}{\partial y}\right|}_{y=0}\text{.}$$

$$C_{f}={\left.{\left.\frac{\mu_{nf}}{\mu_{f}}\frac{\partial V}{\partial y}\right|}_{y=0}=\frac{1}{(1-\phi {)}^{2.5}}\frac{\partial V}{\partial y}\right|}_{y=0},$$

which means that

$$\frac{\mu_{nf}}{\mu_{f}}=\frac{1}{(1-\phi {)}^{2.5}}$$

Discretization of Nusselt number:

$$Nu=-\frac{K_{nf}}{K_{f}}\frac{4T_{1}-T_{2}-3T_{0}}{2h}+o\left(h^{2}\right).$$

Discretization of the Skin friction coefficient

$$C_{f}=-\frac{1}{(1-\phi {)}^{2.5}}\frac{4V_{1}-V_{2}-3V_{0}}{2h}+o\left(h^{2}\right).$$

The discrete problem:

We consider the rectangular domain in space-time Ω = (0 ≤ *ζ* ≤ *L*_Ω_) × (0 ≤ *t* ≤ 1). In the sequel, we shall use the notations Ω_*ζ*_ = [0, *L*_Ω_] for the space domain, and T=[0,1] for the time domain.

Consider a time-space mesh

$$\left(t_{n},\zeta_{j}\right)=\left(t_{0}+l,\zeta_{0}+h\right),\;n\geq 0,\;0\leq j\leq J$$

where J∈N fixed. *l* = *δt* = t_*n*+1_ − *t*_*n*_ is the time step, and *h* = Δ*ζ* = *ζ*_*j*+1_ − *ζ*_*j*_ is the space step.

For a function *F* = *F*(*t*, *ζ*), we apply the approximations

$$F{]}_{j}^{n+1/2}=\frac{1}{2}\left(F_{j}^{n+1}+F_{j}^{n}\right)$$

For the differential operators we shall use the approximations for the first time derivative, and

$${\left.\frac{\partial F}{\partial t}≃\frac{\partial F}{\partial t}\right]}_{j}^{n+1}=\frac{3F^{n+1}-4F^{n}+F^{n-1}}{2l}$$

$${\left.\frac{\partial F}{\partial \zeta }≃\frac{\partial F}{\partial \zeta }\right]}_{j}^{n+1/2}=\frac{1}{2}\left[\frac{F_{j+1}^{n+1}-F_{j-1}^{n+1}}{2h}+\frac{F_{j+1}^{n}-F_{j-1}^{n}}{2h}\right]$$

and

$${\left.\frac{\partial^{2}F}{\partial \zeta^{2}}≃\frac{\partial^{2}F}{\partial \zeta^{2}}\right]}_{j}^{n+1/2}=\frac{1}{2}\left[\frac{F_{j+1}^{n+1}-2F_{j}^{n+1}+F_{j-1}^{n+1}}{h^{2}}+\frac{F_{j+1}^{''}-2F_{j}^{''}+F_{j-1}^{n}}{h^{2}}\right]\text{,}$$

for the first and second order derivatives in space. The numerical solution approximating *F* at the node (*t*_*n*_, *ζ*_*j*_) will be denote by $F_{j}^{n}$. The net function will be in its usual form *F*(*t*_*n*_, *ζ*_*j*_). Finally, we denote $F^{n}=\left(F_{1}^{n},F_{2}^{n},\ldots ,F_{j}^{n}\right)$ the vector composed of the numerical values at the instant *n* on all the space grids. Using these estimations in problem (8) we propose the following discretization,

$$\left\{\begin{array}{l} {\left.{\left.{\left.\frac{\partial V}{\partial t}\right]}_{j}^{n+1}=a_{1}\frac{\partial^{2}V}{\partial \zeta^{2}}\right]}_{j}^{n+1/2}+a_{2}T\right]}_{j}^{n+1/2}\\ {\left.{\left.{\left.\frac{\partial T}{\partial t}\right]}_{j}^{n+1}=a_{3}\frac{\partial^{2}T}{\partial \zeta^{2}}\right]}_{j}^{n+1/2}+a_{4}\frac{\partial }{\partial \zeta }\left(T^{3}\frac{\partial T}{\partial \zeta }\right)\right]}_{j}^{n+1/2} \end{array}\right.$$ (10)

where the nonlinear term $\frac{\partial }{\partial \zeta }\left(T^{3}\frac{\partial T}{\partial \zeta }\right)$ is estimated by

$${\left.\frac{\partial }{\partial \zeta }\left(T^{3}\frac{\partial T}{\partial \zeta }\right)\right]}_{j}^{n+1/2}=\frac{{\left(T_{j+1/2}^{n}\right)}^{3}T_{j+1}^{n+1}-\left[{\left(T_{j+1/2}^{n}\right)}^{3}+{\left(T_{j-1/2}^{n}\right)}^{3}\right]T_{j}^{n+1}+{\left(T_{j-1/2}^{n}\right)}^{3}T_{j-1}^{n+1}}{h^{2}}$$

where

$$T_{j}^{n+1/2}=\frac{T_{j+1}^{n}+T_{j}^{n}}{2},\;\text{and}\;T_{j}^{n-1/2}=\frac{T_{j}^{n}+T_{j-1}^{n}}{2}$$

Denote for the next

$$\sigma =\frac{l}{h^{2}}=\frac{\delta t}{\Delta \zeta^{2}}$$

By replacing each discrete estimation by its exact expression, we derive from the system (10) the following discrete form

$$\left\{\begin{array}{l} -a_{1}{\sigma V}_{j-1}^{n+1}+\left(3+2a_{1}\sigma \right)V_{j}^{n+1}-a_{1}{\sigma V}_{j+1}^{n+1}-a_{2}{lT}_{j}^{n+1}\\ =a_{1}{\sigma V}_{j-1}^{n}+\left(4-2a_{1}\sigma \right)V_{j}^{n}+a_{1}{\sigma V}_{j+1}^{n}-V_{j}^{n-1}+a_{2}{lT}_{j}^{n}\\ -\left(a_{3}+2a_{4}{\left(T_{j-1/2}^{n}\right)}^{3}\right){\sigma T}_{j-1}^{n+1}+\left(3+2\left(a_{3}+a_{4}\left[{\left(T_{j+1/2}^{n}\right)}^{3}+{\left(T_{j-1/2}^{n}\right)}^{3}\right]\right)\sigma \right)T_{j}^{n+1}\\ -\left(a_{3}+2a_{4}{\left(T_{j+1/2}^{n}\right)}^{3}\right){\sigma T}_{j+1}^{n+1}\;=a_{3}{\sigma T}_{j-1}^{n}+\left(4-2a_{3}\sigma \right)T_{j}^{n}+a_{3}{\sigma T}_{j+1}^{n}-T_{j}^{n-1}. \end{array}\right.$$ (11)

The system (11) is considered under the initial, and the artificial boundary values conditions

$$\left\{\begin{array}{l} V^{0}=0\;\text{and}\;T^{0}=(1,0,0,\ldots ,0)\\ V_{0}^{n}=V_{J}^{n}=0\;\text{and}\;T_{0}^{n}=1,T_{J}^{n}=0,\;\forall n\geq 1 \end{array}\right.$$

We then obtain tridiagonal linear systems which will be solved by the classical algorithm of Thomas.

After determining the velocity and temperature fields, it becomes possible to calculate the Nusselt number N_u_ and the skin friction coefficient *C*_*f*_ by the following expressions:

$$Nu=-\frac{K_{nf}}{K_{f}}\frac{4T_{1}-T_{2}-3T_{0}}{2h}+o\left(h^{2}\right).$$

$$C_{f}=-\frac{1}{(1-\phi {)}^{2.5}}\frac{4V_{1}-V_{2}-3V_{0}}{2h}+o\left(h^{2}\right).$$

## 3. Validation

**Fig 2:**
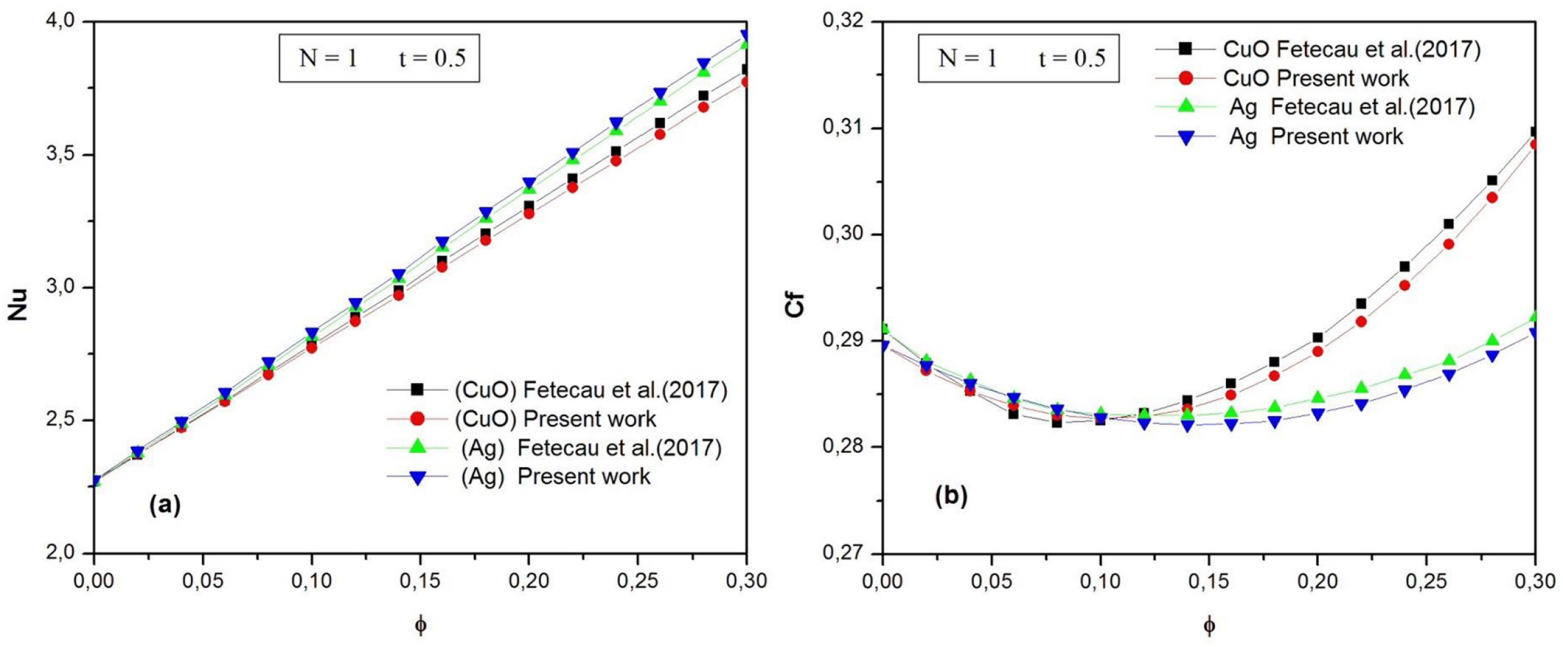
The Nusselt number Nu (a) and the skin friction coefficient C_f_ (b) against the nanoparticle.

To demonstrate the accuracy of the current results, a comparison was made with existing results in the literature. The validation test focuses on the numerical results presented by Fetecau et al. [19] concerning the natural convection flow of nanofluids along an infinite vertical plate with thermal radiation. Two types of nanofluids were used: CuO-water and Ag-water. Initially, the plate and fluid are at rest and at ambient temperature *T*_*∞*_. Suddenly, the plate temperature rises to a constant value *T*_*w*_ (*T*_*w*_ > *T*_*∞*_), generating natural convection flow. Fig 2a shows the variation of the Nusselt number as a function of the nanoparticle volume fraction *φ* for the two water-based nanofluids when radiation number N = 1 and time t = 0.5. The present results are in good agreement for both nanoparticles.

The variation of the skin friction coefficient C_f_ with nanoparticle volume fraction *φ* for CuO-water and Ag-water nanofluids when radiation number N = 1 and time t = 0.5 is displayed in Fig 2b. As can be seen, our results are in excellent agreement with the numerical results presented by Fetecau et al. [19].

## 4. Results and discussion

Numerical calculations were carried out to investigate the impact of different parameters such as time, thermal radiation, and the volume fraction of nanoparticles on the natural convection flow of nanofluids along a heated plate with thermal radiation. The radiation parameter N is considered in the range of 0 to 2, while the volume fraction of nanoparticles *φ* is varied between 0 and 0.12. The Prandtl number *Pr* of the fluid is taken equal to 6.2.

### 4.1 CuO-water nanofluid

The study starts by focusing on the case of the CuO-water nanofluid to analyze the effects of nonlinearity and relevant parameters on temperature and velocity fields, heat transfer rate, and skin friction coefficient. Then, the analysis is extended to four other types of nanofluids. The results of the study will be presented graphically.

**Fig 3:**
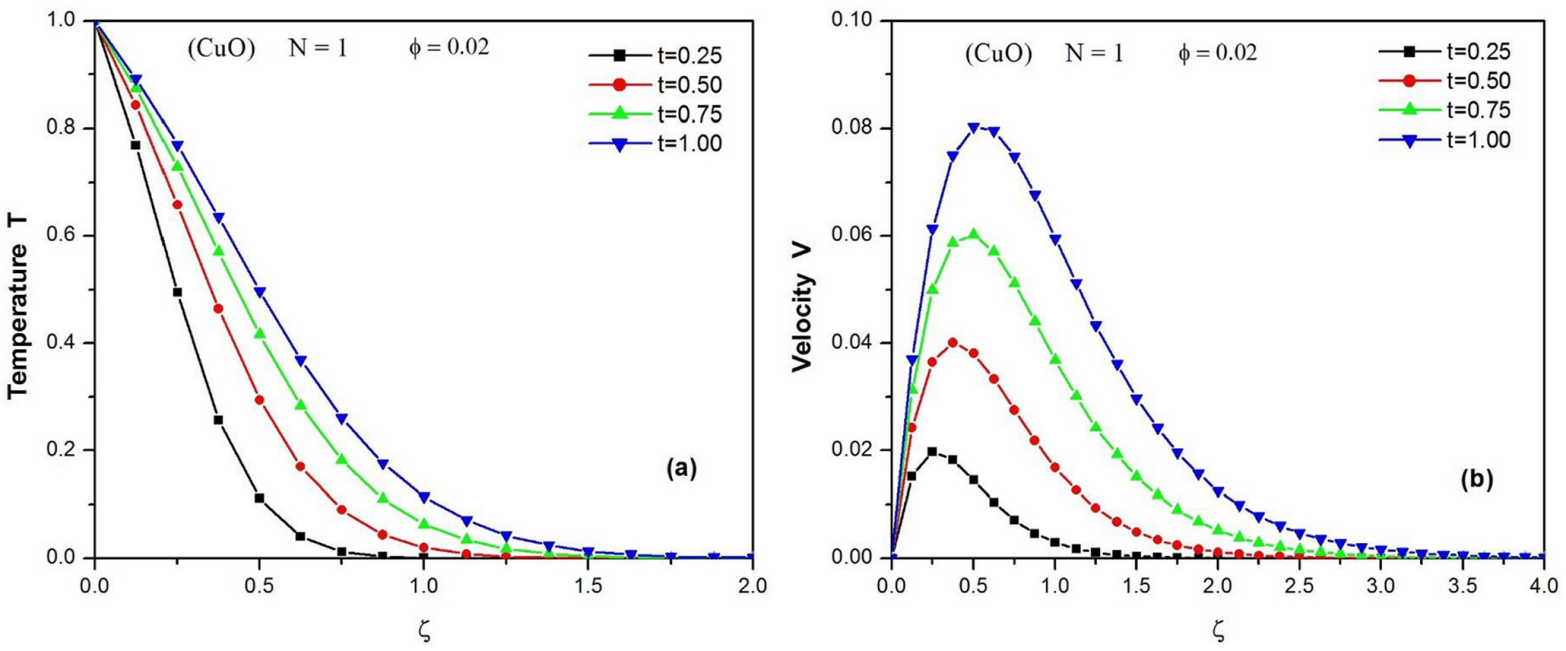
Temperature (a) and Velocity (b) profiles of CuO water nanofluid at different values of t (0.25 ≤ *t* ≤ 1.) when N = 1 and ϕ = 0.02.

Fig 3(a) shows the temporal variation of the temperature profiles of the CuO water nanofluid when N = 1 and *φ* = 0.02. The profiles are decreasing curves where the temperature goes from T = 1 at the level of the wall to T = 0 far from it. The figure also reveals that when time progresses, the temperature of the fluid T increases, which means that the thermal boundary layer becomes thicker.

Fig 3(b) illustrates the evolution of the velocity profiles of the CuO water nanofluid over time when N = 1 and *φ* = 0.02. The fluid velocity is zero close to the plate and increases under the effect of buoyancy forces, reaching its maximum speed before slowly decreasing to zero far from the wall. It can be noted that over time the momentum boundary layer thickens.

**Fig 4:**
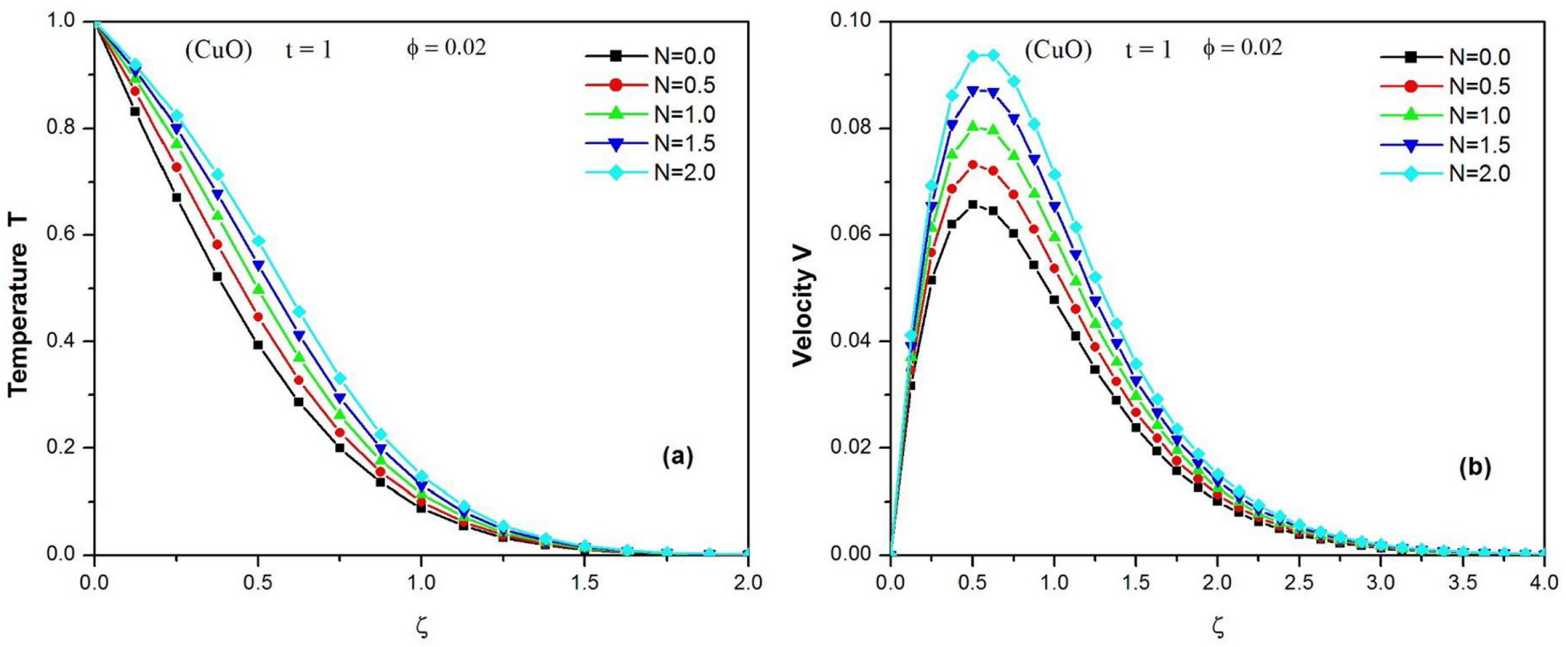
Temperature (a) and Velocity (b) of CuO water nanofluid at different values of N (0. ≤ *N* ≤ 2.) when t = 1 and ϕ = 0.02.

Fig 4(a) and 4(b) show the effect of the thermal radiation parameter (N) on the temperature and velocity profiles of the CuO-water nanofluid when t = 1 and *φ* = 0.02, respectively. It should be emphasized that the temperature and the velocity of the nanofluid increase with the thermal radiation parameter. It will also be noted that a decrease in the rate of radiative heat transfer to the fluid, favors an increase in the fluid speed.

**Fig 5:**
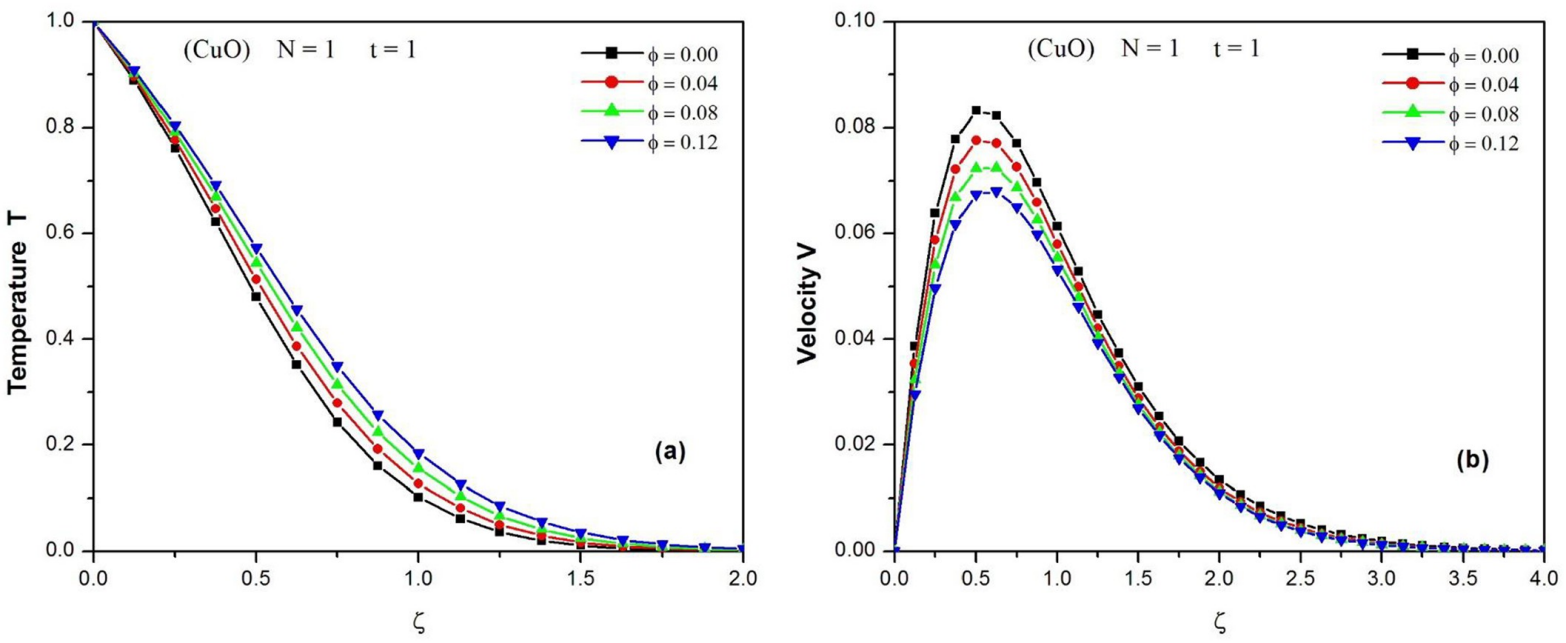
Temperature (a) and Velocity (b) of CuO-water nanofluid at different values of φ (0. ≤ *ϕ* ≤ 0.12) when N = 1 and t = 1.

Fig 5(a) demonstrates the effect of the nanoparticle volume fraction *φ* on the temperature profiles of copper oxide (CuO)-water nanofluid when N = 1 and t = 1. It is observed that the temperature of the nanofluid decreases when moving away from the plate and increases when the volume fraction of the nanoparticles increases. After a time, t = 1, the temperature goes from T = 1 in the vicinity of the plate to T = 0 for *ζ* ≥ 2. The increase in temperature can be explained by an improvement of the thermal conductivity of the nanofluid due to the increase in the volume fraction of the nanoparticles, which also leads to an increase in the thickness of the thermal boundary layer.

The effect of the volume fraction *φ* of the nanoparticles on the velocity profiles of the copper oxide (CuO)-water nanofluid is shown in Fig 5(b) when N = 1 and t = 1. From this figure it can be concluded that the velocity of the nanofluid decreases when the volume fraction of the nanoparticles increases. The velocity profiles have the same shape. Upon contact with the plate, the fluid velocity is very low. Due to buoyancy forces, the fluid accelerates and its velocity increases until it reaches a maximum at about ζ = 0.5, then slowly decreases until it cancels at ζ = 3.5. The increase in the volume fraction of the nanoparticles causes a reinforcement of the viscous forces, leading to a slowing down of the flow.

**Fig 6:**
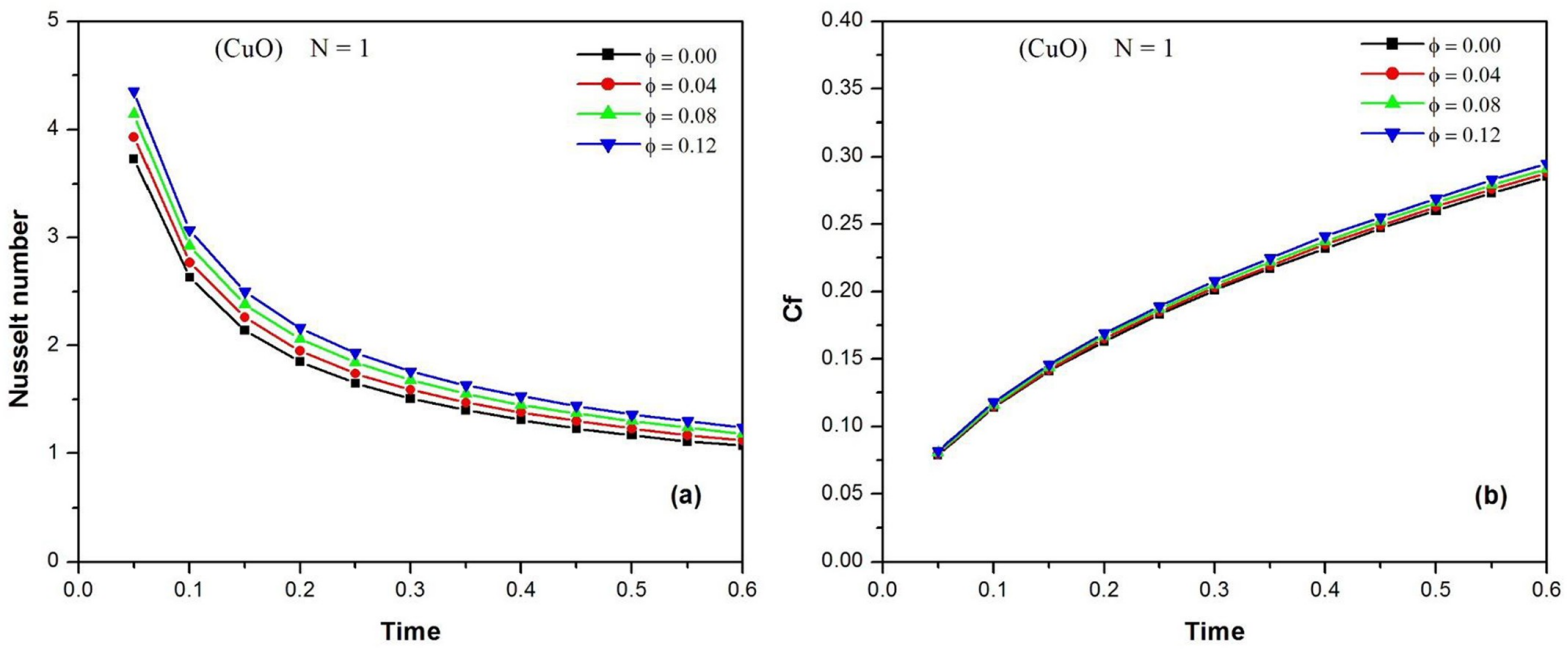
Time variation of the Nusselt number (a) and the skin friction coefficient C_f_ (b) of CuO-water nanofluid at different values of φ (0. ≤ *ϕ* ≤ 0.12) when N = 1.

**Fig 7:**
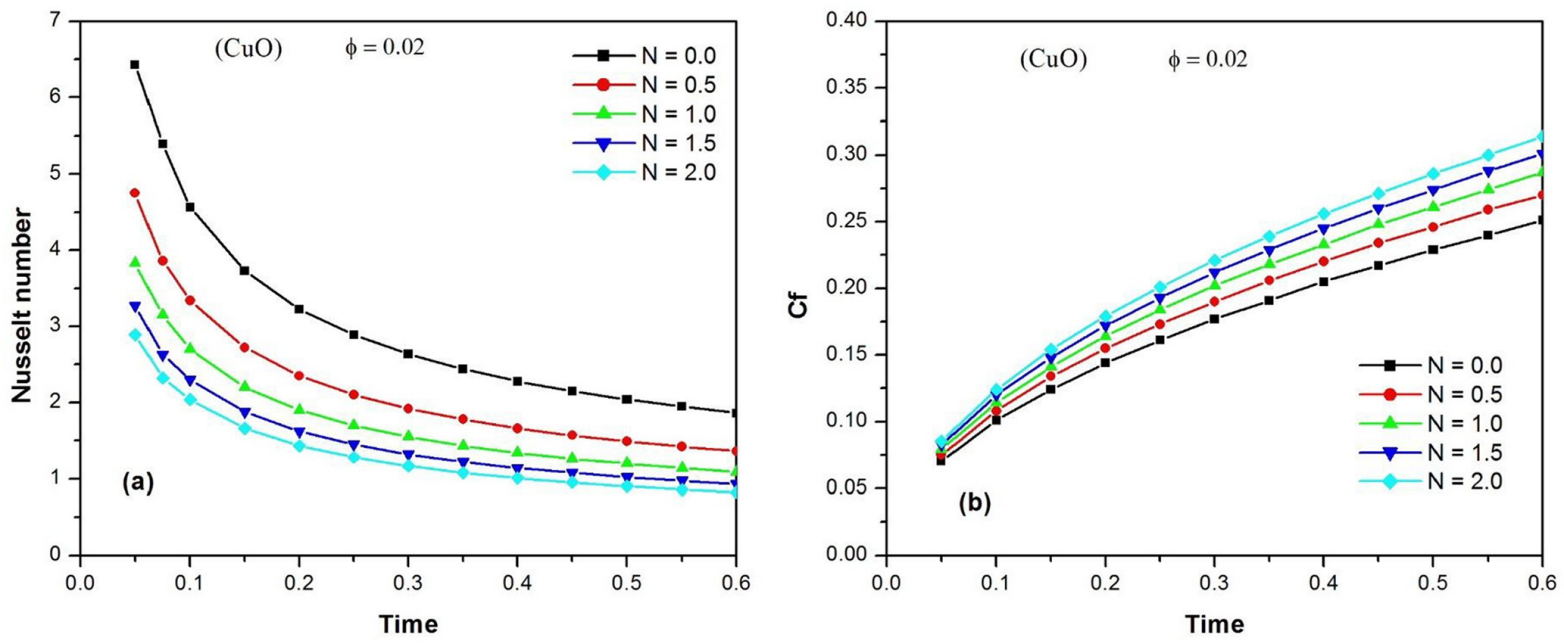
Time variation of the Nusselt number (a) and the skin friction coefficient C_f_ (b) of CuO-water nanofluid for different values of N (0. ≤ *N* ≤ 2.) when ϕ = 0.02.

Fig 6(a) illustrates the time variation of the Nusselt number of CuO water nanofluid at different values of *φ* when N = 1. It is observed that the increase in the volume fraction of the nanoparticles enhances the heat transfer rate. The time variation of the skin friction coefficient Cf of the CuO water nanofluid at different values of the volume fraction φ of the nanoparticles is shown in Fig 6(b), when N = 1. It is noted that increasing the skin friction (or shear stress) is typically a negative aspect in engineering applications. From the figure, it can be concluded that the skin friction Cf increases over time, and that an increase in the volume fraction of the nanoparticles increases the shear stress. In order to highlight the effect of the radiation parameter N on the heat transfer rate, the temporal variation of the Nusselt number of CuO water nanofluid for different values of N is presented in Fig 7(a), when *φ* = 0.02.The Nusselt number decreases rapidly with time and tends towards stable values. It is also observed that increasing the thermal radiation parameter N improves the heat transfer rate. Fig 7(b) depicts the effect of the radiation parameter N on the shear stress by displaying the time variation of the skin friction coefficient C_f_ of the CuO-water nanofluid for different values of N when φ = 0.02. It can be seen that the skin friction coefficient C_f_ increases over time and the effect of radiation on the shear stress becomes more visible with significant deviations between the curves. It is also observed that an increase in the thermal radiation parameter N increases the skin friction coefficient C_f_, which is an unfavorable situation for technological applications.

### 4.2 Effect of nonlinearity

In order to clarify the effect of nonlinearity on the physics of the problem, a comparative study is conducted to evaluate the deviations between the numerical solutions of temperature, velocity, heat transfer rate and shear stress when the linear or nonlinear Rosseland approximation is used to model the radiative term.

**Fig 8:**
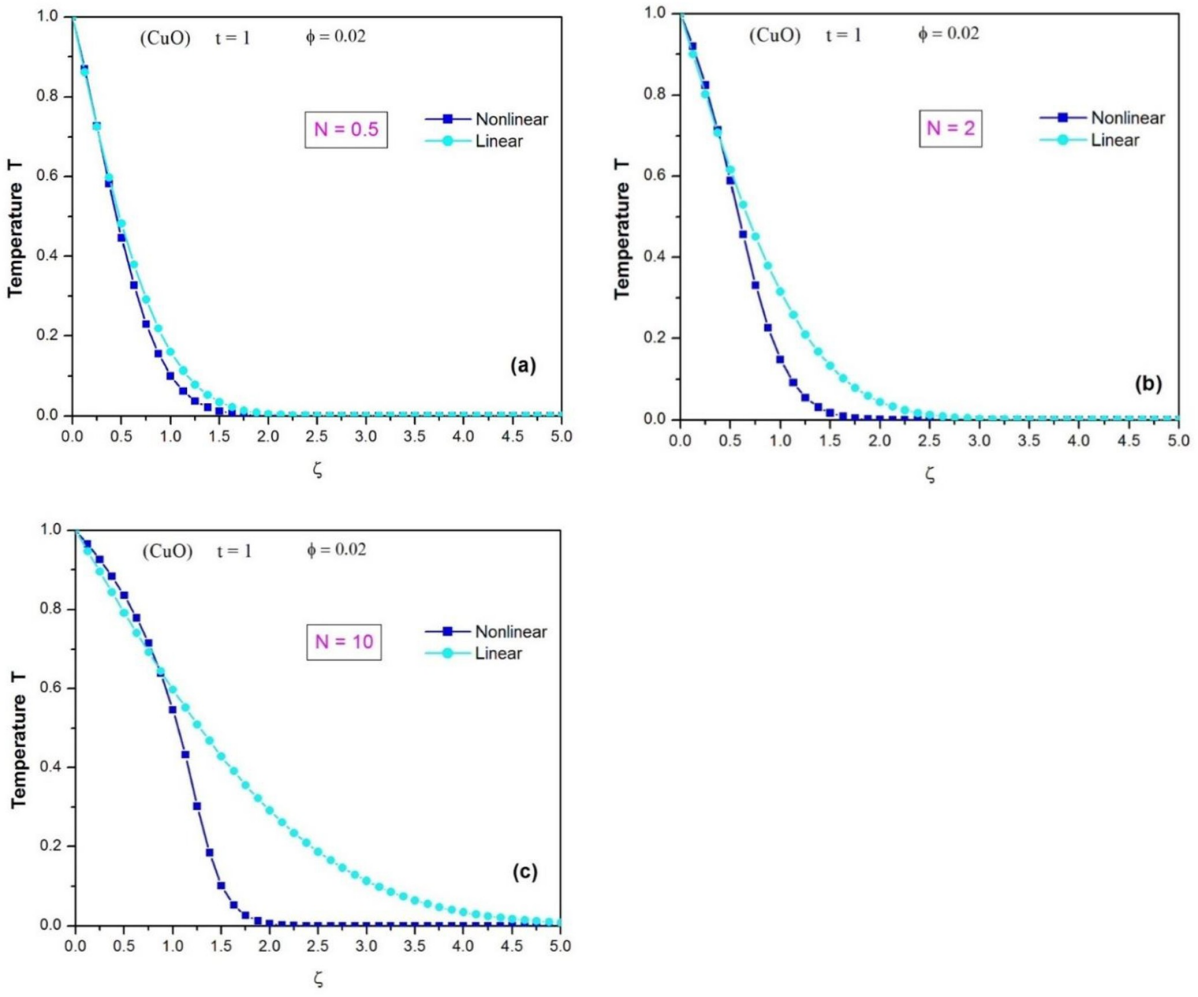
Comparison of the temperature profiles of the CuO water nanofluid using the linear and nonlinear Rosseland approximations (when ϕ = 0.02 and t = 1), (a) for N = 0.5; (b) for N = 2; (c) for N = 10.

Fig 8(a) displays the temperature profiles of the CuO-water nanofluid using the linear and nonlinear Rosseland approximations when φ = 0.02 and t = 1. When the effect of thermal radiation is small (N = 0.5), the temperature profiles are similar, which means that the linear and nonlinear Rosseland approximations give almost the same results as shown in Fig 6(a).

Fig 8(b) depicts the temperature profiles of CuO water nanofluid when the thermal radiation impact is moderate (N = 2). Far from the heated wall, the differences between the two temperature profiles become significant, which means that the linear approximation of Rosseland has exceeded its limit of validity.

When N = 10 (high thermal radiation), the differences between the temperature profiles are significant, and it can be seen that the linear Rosseland approximation is no longer valid as shown in Fig 8(c). It’s also worth noting that the thickness of the thermal boundary layer increases slightly with N, when using the non-linear Rosseland approximation, but the linear approximation of the radiative term amplifies the thickness of the thermal boundary layer.

**Fig 9:**
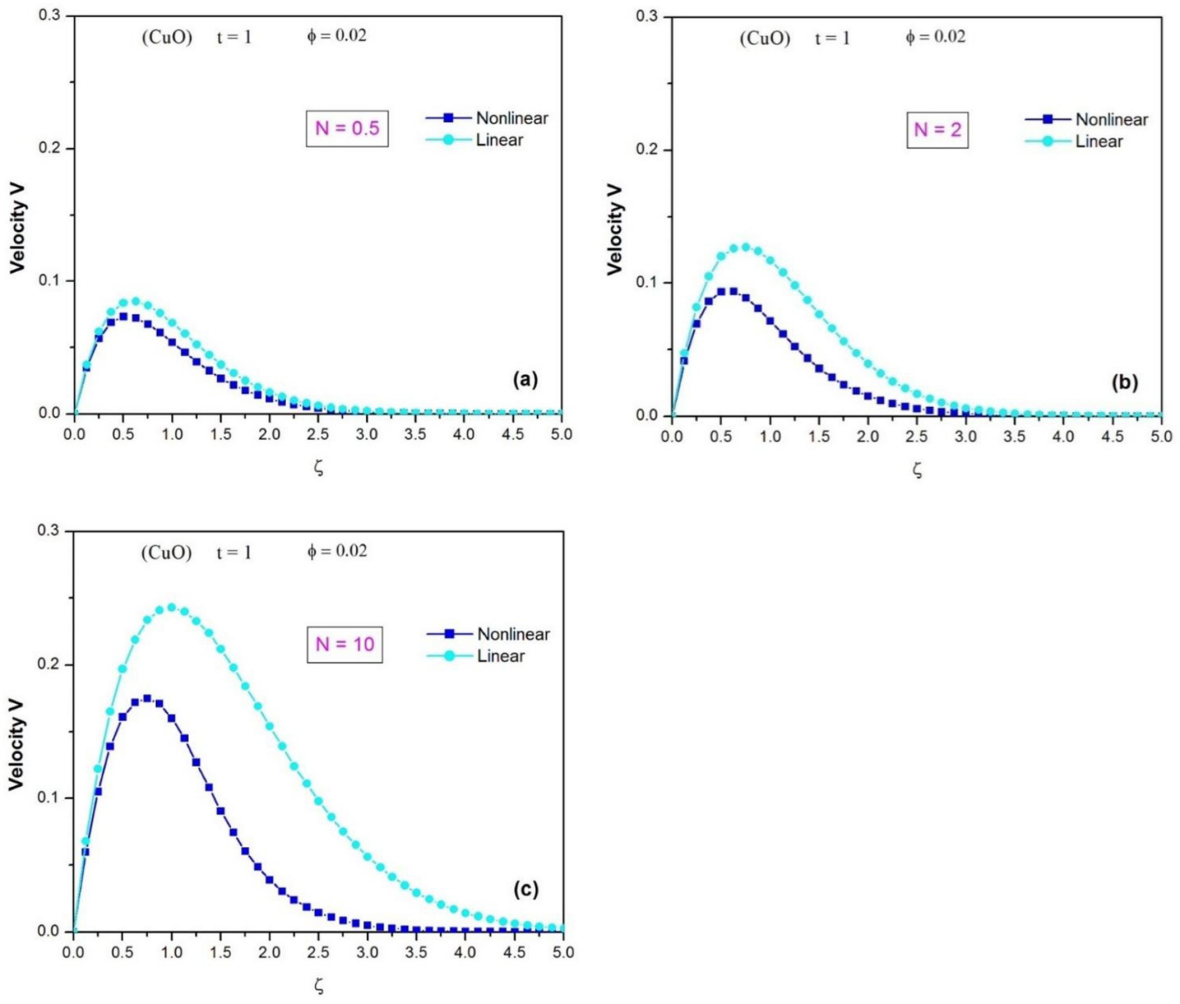
Comparison of the velocity profiles of the CuO water nanofluid using the linear and nonlinear Rosseland approximations (when ϕ = 0.02 and t = 1), (a) for N = 0.5; (b) for N = 2; (c) for N = 10.

Fig 9(a) illustrates the velocity profiles of the CuO water nanofluid using both the linear and nonlinear Rosseland approximations, when *φ* = 0.02 and t = 1. When the thermal radiation effect is weak, as seen in Fig 9(a), the velocity profiles of the two approximations are similar and very close, indicating that the linear and nonlinear Rosseland models give similar results at low values of the thermal radiation parameter (N = 0.5). However, when the impact of thermal radiation is moderate (N = 2), as can be seen in Fig 9(b), there are significant differences between the two velocity profiles. In particular, the maximum fluid velocity is overestimated by the linear Rosseland model, making it invalid. The same conclusion is drawn in Fig 9(c) when the effect of thermal radiation is high (N = 10), the linear Rosseland approximation also overestimates the maximum fluid velocity and the thickness of the momentum boundary layer.

**Fig 10:**
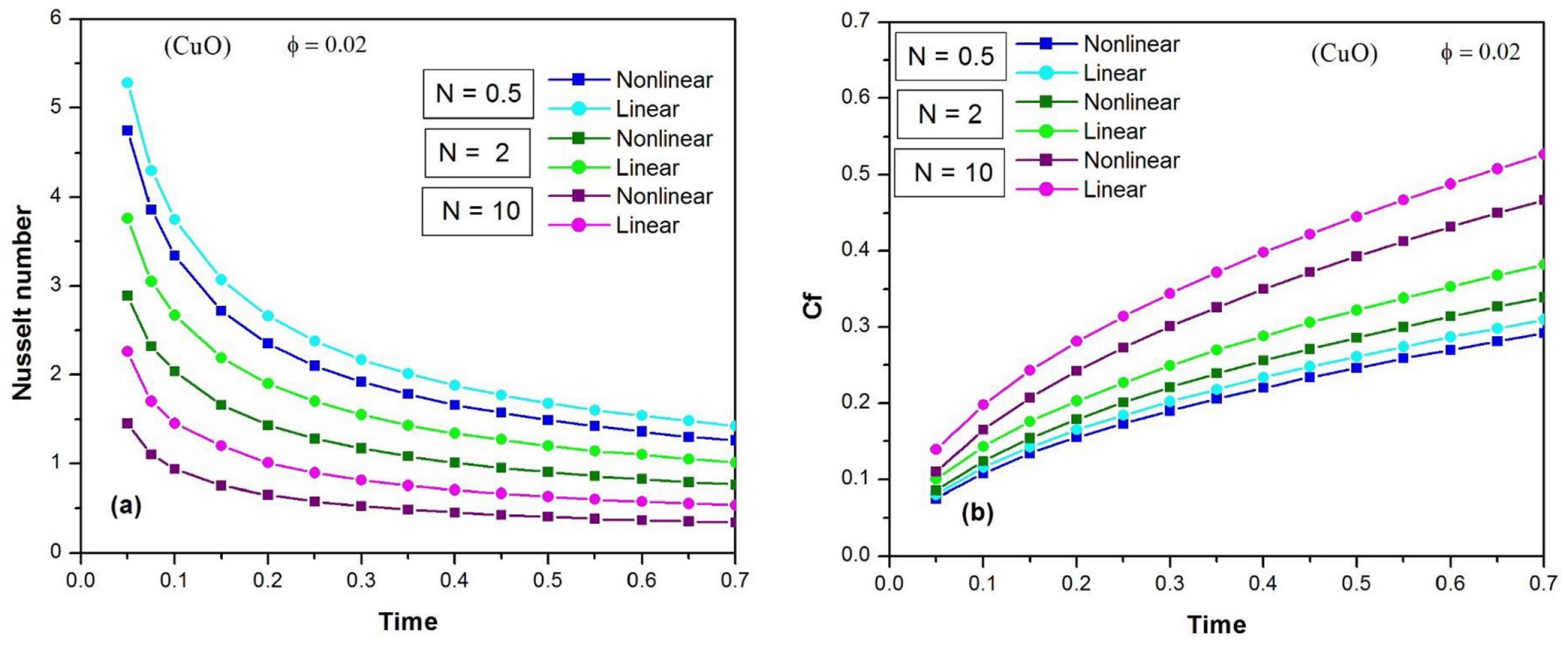
Time variation of the Nusselt number (a) and the skin friction coefficient C_f_ (b) of CuO water nanofluid using the linear and nonlinear Rosseland approximations for different values of N when ϕ = 0.02.

The heat transfer rate at the heated vertical plate is also an important factor for engineering purposes. Fig 10(a) shows the changes in the Nusselt number of a CuO water nanofluid over time using linear and nonlinear Rosseland approximations for different values of N, when *φ* = 0.02. The Nusselt number decreases over time, indicating that the heat transfer rate decreases and eventually reaches a stable value in the case of transient natural convection flow. In general, the Nusselt number decreases as the thermal radiation parameter N increases. It should be noted that the Rosseland linear approximation overestimates the heat transfer rate.

In order to evaluate the impact of nonlinearity on the shear stresses, Fig 10(b) describes the time variation of the skin friction coefficient of the CuO water nanofluid over time using both linear and nonlinear Rosseland approximations for various values of N, when *φ* = 0.02. The skin friction coefficient increases over time for the transient natural convection flow. As the thermal radiation parameter N increases, the effect of shear stresses is intensified. It is worth noting that the linear approximation of Rosseland overestimates the skin friction coefficient.

In summary, it can be stated that the Rosseland linear approximation is accurate for small values of the thermal radiation parameter N. However, when the effect of thermal radiation is large, the Rosseland linear model overestimates the temperature and velocity of the fluid, and consequently, the thicknesses of the thermal and momentum boundary layers. In addition, the heat transfer rate and the skin friction coefficient C_f_ are also overestimated. In such cases, the use of the nonlinear Rosseland approximation is more appropriate.

It should be stressed that the detailed study of the variation of velocity and temperature fields as a function of different parameters has been limited to the case of the CuO-water nanofluid, in order not to overload the present study. However, it should be noted that similar calculations undertaken for other nanoparticles (Ag, Al_2_O_3_, Cu and TiO_2_) showed similar behavior for all nanofluids, albeit at different scales.

### 4.3 Performance comparison of nanofluids

In this section, the performance of five types of nanofluids containing CuO, Ag, Al_2_O_3_, Cu and TiO_2_ will be compared using the average Nusselt number N_u_ and the average skin friction coefficient C_f_ as benchmark. These quantities of technical interest are time averages calculated over a period of *τ* = 1 when N = 1 and for a nanoparticle volume fraction *φ* ranging from 0 to 0.12.

**Fig 11:**
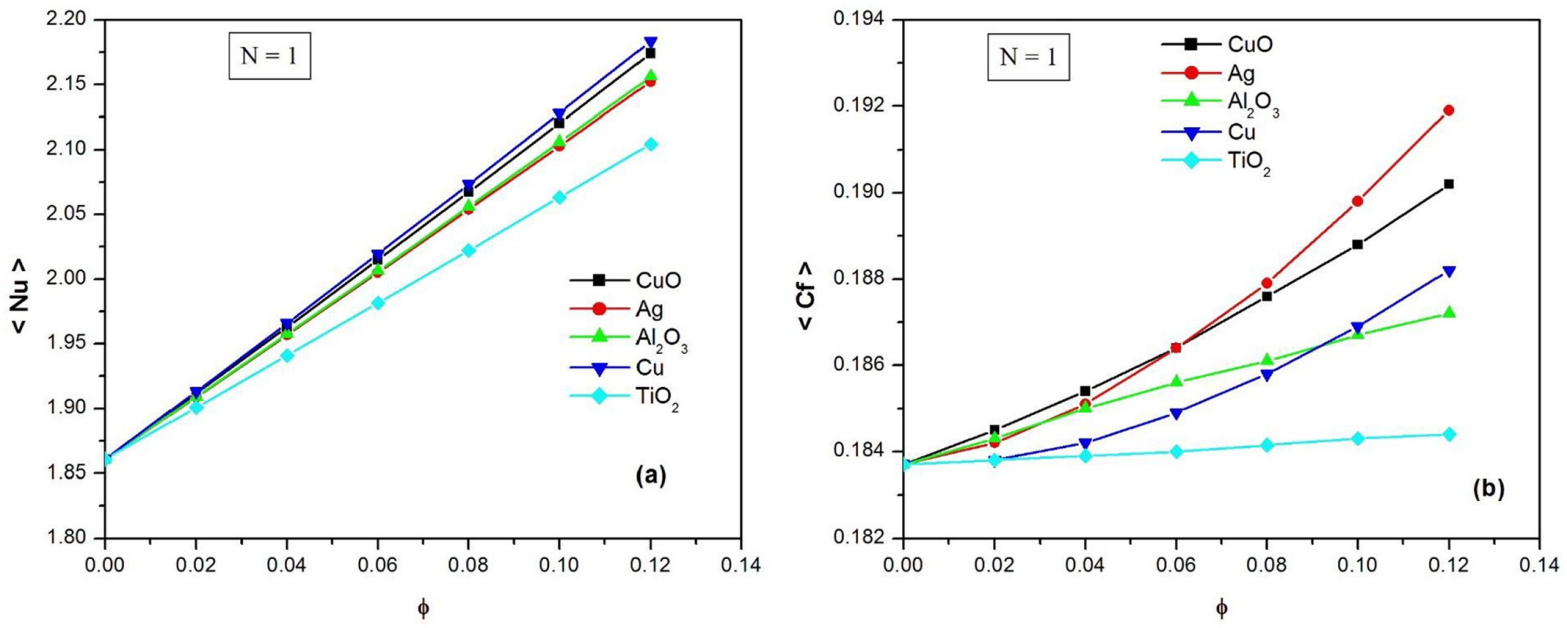
The average Nusselt number <Nu> (a) and the average skin friction coefficient < C_f_ > (b) against the nanoparticle volume fraction ϕ for five water-based nanofluids using the nonlinear Rosseland approximation when N = 1.

Fig 11(a) illustrates the variation of the average Nusselt number against the nanoparticle volume fraction *φ* for the five water-based nanofluids using the nonlinear Rosseland approximation, when N = 1. This figure reveals that the heat transfer rates Nu of the nanofluids are nearly straight lines and increase linearly with *φ*. For small values of the volume fraction *φ*, the Nusselt numbers of the different nanofluids are quite similar. However, as *φ* increases, the curves diverge. It was observed that the highest heat transfer occurs for Cu and CuO, which have very close curves. On the other hand, the lowest heat transfer occurs for TiO_2_ nanoparticles. It is worth noting that the heat transfer rates of the water Ag and Al_2_O_3_ nanofluids are identical, despite Ag having the highest thermal conductivity.

Fig 11(b) shows the variations of the average skin friction coefficient C_f_ as a function of nanoparticle volume fraction *φ* for five nanofluids using the nonlinear Rosseland approximation, when N = 1. It is observed that the skin friction coefficient C_f_ increases monotonically as a function of *φ*. It is worth noting that the increase of C_f_ is linear for Al_2_O_3_ and TiO_2_ and nonlinear for CuO, Ag and Cu. It was found that the highest shear stresses are for CuO when *φ* ≤ 0.06 and for Ag when *φ* ≥ 0.06. On the other hand, it should be noted that the lowest shear stress occurs for TiO_2_ nanoparticles. To summarize, it can be concluded that among the five nanofluids, Cu performs the best by providing the highest heat transfer rate with a relatively low skin friction coefficient.

## 5. Conclusion

An original finite-difference method is proposed to study the effects of thermal radiation nonlinearity on the natural convection flow of a nanofluid along an infinite heated plate at constant temperature. The ultimate goal is to improve the performance of flat and curved photovoltaic modules integrated into buildings using nanofluids. Unlike conventional approaches that linearize the highly nonlinear radiative term, our model directly solves the nonlinear problem using Rosseland’s nonlinear approximation with second-order accuracy. After validation of the numerical method, the effects of relevant physical parameters such as time, volume fraction and radiation parameters on the velocity, temperature, heat transfer rates and shear stresses of the CuO-water nanofluid were determined and analyzed. Following a discussion of the effect of non-linearity, the analysis was extended to other nanoparticles (Ag, Al_2_O_3_, Cu and TiO_2_).

The study found that:

- Thermal radiation significantly affects nanofluid temperature and velocity.
- As the time and thermal radiation parameter N increase, the nanofluid velocity and temperature increase.
- Increasing the volume fraction φ of the nanoparticles raises the temperature of the nanofluid while decreasing its velocity.
- An increase in the thermal radiation parameter N decreases the rate of heat transfer and amplifies skin friction coefficients, which hampers technical applications.
- Increasing the volume fraction φ improves the heat transfer rate, although it increases viscous forces and surface shear stresses.
- When the effect of thermal radiation is significant, Rosseland’s linear approximation becomes invalid and leads to an overestimation of temperature, fluid velocity, heat transfer rate and skin friction coefficient C_f_. In this case, only the non-linear formulation is capable of reflecting the physical reality of radiative exchanges.
- For all five nanoparticle types (CuO, Ag, Al2O3, Cu and TiO2) the average Nusselt number and skin friction coefficient increase with volume fraction φ.
- The Cu-water nanofluid is the best-performing of the five nanofluids, with a high heat transfer rate and low skin friction coefficient.

## Supporting information

S1a Fig Origin data for Fig 1a. (OPJ)

S1b Fig Origin data for Fig 1b. (OPJ)

S2a Fig Origin data for Fig 2a. (OPJ)

S2b Fig Origin data for Fig 2b. (OPJ)

S3a Fig Origin data for Fig 3a. (OPJ)

S3b Fig Origin data for Fig 3b. (OPJ)

S4a Fig Origin data for Fig 4a. (OPJ)

S4b Fig Origin data for Fig 4b. (OPJ)

S5a Fig Origin data for Fig 5a. (OPJ)

S5b Fig Origin data for Fig 5b. (OPJ)

S6a Fig Origin data for Fig 6a. (OPJ)

S6b Fig Origin data for Fig 6b. (OPJ)

S7a Fig Origin data for Fig 7a. (OPJ)

S7b Fig Origin data for Fig 7b. (OPJ)

S7c Fig Origin data for Fig 7c. (OPJ)

S8a Fig Origin data for Fig 8a. (OPJ)

S8b Fig Origin data for Fig 8b. (OPJ)

S8c Fig Origin data for Fig 8c. (OPJ)

S9a Fig Origin data for Fig 9a. (OPJ)

S9b Fig Origin data for Fig 9b. (OPJ)

S10a Fig Origin data for Fig 10a. (OPJ)

S10b Fig Origin data for Fig 10b. (OPJ)
